# Successful transposition of sperm freezing, in vitro fertilization and artificial insemination methodologies in the Tadjik markhor species (*Capra falconeri heptneri*)

**DOI:** 10.1590/1984-3143-AR2024-0071

**Published:** 2024-08-12

**Authors:** Yann Locatelli, Colin Vion, Nicolas Duffard, Nicolas Bon, Jérémy Bernard, Charly Binaud, Nina Trontti, Gérard Baril, Pascal Mermillod

**Affiliations:** 1 Muséum National d’Histoire Naturelle, Laboratoire de la Réserve Zoologique de la Haute Touche, Obterre, France; 2 Institut National de Recherche pour l’Agriculture, l’Alimentation et l’Environnement, Centre National de la Recherche Scientifique, Université de Tours, Nouzilly, France; 3 Helsinki Zoo Korkeasaari, Helsinki, Finlande

**Keywords:** semen, artificial insemination, embryo production, LOPU

## Abstract

The objective of the present study was to transpose sperm freezing methodology from domestic goat to the Tadjik markhor (*Capra falconeri heptneri*) and to address the feasibility to develop IVP and artificial insemination using such frozen semen. Semen of different adult markhor males were successfully recovered by electro-ejaculation and were then frozen using caprine methodology. Frozen semen showed good survival rates at thawing and good fertility rates were assessed in heterologous *in vitro* fertilization system with goat oocytes. LOPU/IVF was applied for Tadjik markhor females allowing the first successful blastocyst production *in vitro*. In an applied program, we also transposed successfully intrauterine AI method with frozen/thawed semen to the Tadjik markhor.

## Introduction

The extinction rate of animal species has increased sharply in recent decades, leading to a major loss of biodiversity. When measures are insufficient in nature, *ex situ* conservation represents an alternative nowadays realized worldwide in zoos or reserves. *Ex situ* conservation programs have limits, particularly in terms of maintaining genetic diversity. To improve the success of these programs, the use of biological resource banks and assisted reproductive technologies has been proposed (Locatelli, 2024). As part of the development of a conservation program within the EAZA (European association of Zoos and Aquaria), a study was designed to promote the preservation of genetic diversity in the markhor goat (*Capra falconeri heptneri*) by implementing assisted reproduction techniques for markhor species. Amongst objectives identified, creation of a biobank of frozen semen for this species to guarantee long-term storage of this precious genetics was first designed. The second objective was to set up of the artificial insemination technique with frozen semen, to allow the dissemination of genetic between conservation sites and facilitate husbandry recommendations. Development of *in vitro* fertilization methods and interspecific embryo transfer techniques was the third objective.

## Methods

All experiments were carried out in accordance with the procedural and ethical authorization of the French Government (DDCSPP authorization number C-36-145-002 to Réserve Zoologique de La Haute Touche). The procedures and ethical consideration regarding a non-experimental animal model (*Capra falconeri heptneri*) were submitted to evaluation and approved by the EAZA Caprinae TAG committee.

### Sperm recovery, cryoconservation and viability evaluation

Semen from different adult markhor males was collected by electro-ejaculation on Réserve Zoologique de la Haute Touche. These samples were carried out opportunistically between 2005 and 2009 during the breeding season after anaesthesia necessary for animal transfers or veterinary care. Shortly after anaesthesia, animals were submitted to electroejaculation protocol using Minitube Electroejaculator 11900 (Minitube®, Tiefenbach, Germany) and 1“ probe following stimulation Curve 0 (0.5 to 16V, 2.5 s TON or pulse time, 3.5 s TOF or interval time). Procedure was performed twice in case of absence of ejaculate at first trial. Ejaculates were recovered in pre warmed artificial vagina and samples were processed immediately after recovery for evaluation and cryopreservation. Anaesthesia was reversed after the end of the foot care procedure using Atipamezole administered IM.

The collected semen from 6 males was cryopreserved according to the method developed for domestic goat (Baril et al., 1993). Two samples of 10 µl of freshly collected semen were taken to determine massal (0-5), total and individual motilities and sperm concentration of the ejaculate. Double washing of the seminal plasma was performed immediately after collection. The first wash was carried out at 30°C by suspending the sperm in a washing solution (Krebs-Ringer-Phosphate KRPG) containing glucose as described previously (Corteel and Baril, 1974). Diluted ejaculate (400x10^6^ sptz/mL) was then centrifugated at 600 x g for 15 min at RT to eliminate the seminal plasma. Supernatant was removed after centrifugation, and procedure was repeated. The total number of spermatozoa available from the ejaculate was reduced by 15% during the procedure. After washing, the spermatozoa were immediately pre-diluted at 20°C, in a milk diluent (100 g/L skimmed milk from cow) supplemented with Glucose (1,94 g/L) and Gentamicin (20 µg/mL), at concentration of 400x10^6^ spermatozoa/mL. The temperature of the semen was decreased thereafter at a rate of 0.2°C/min to reach 4°C. At 4°C, extender 2 (extender 1 supplemented with 14% glycerol) was added 1:1 in 4 successive fractions at 5 minutes interval. Sperm was frozen in 2 steps in nitrogen vapor. At thawing, sperm was resuspended in SOFaa (Synthetic Oviduct Fluid) (Souza-Fabjan et al., 2014) supplemented with BSA (1mg/mL) and Hepes (2,38 mg/mL) for evaluation. Motile and progressive sperm proportions, STR, LIN as well as velocitiy parameters (VSL, VCL, VAP and ALH) were assessed at 5, 10 and 60 min after thawing by an automated system (CASA Hamilton Thorne).

### Heterologous in vitro fertilization

In order to evaluate the fertility of the frozen semen of the 6 markhor males, domestic goat oocytes were recovered from slaughterhouse ovaries in order to perform heterologous IVF. Oocytes were aspirated from follicles on goat ovaries, selected and in vitro matured as described previously (Souza-Fabjan et al., 2016, 2014). For IVF, selected COCs were co-incubated with markhor spermatozoa according to a protocol set up for domestic goats (IVF SOFaa in the presence of 10% estrous sheep serum, SS). Motile spermatozoa from each male were selected using centrifugation of the thawed semen (15 min at 700 x g) on a Percoll density gradient (45/90%). Sperm pellets were resuspended in SOFaa supplemented with Hepes and centrifuged for 5 min at 100 x g. The sperm pellet was diluted to 10x10^6^ spermatozoa/mL in SOFaa supplemented with 10% SS for 10 min. Motile spermatozoa were co-incubated with oocytes for 18 h at a final concentration of 2x10^6^ spermatozoa/mL at 38.8 °C, in humidified atmosphere of 5% CO2 in air. The hybrid embryos produced were cultured *in vitro* at 38.8°C, in humidified atmosphere in 25µL microdroplets in SOFaa supplemented with BSA and 10% FCS at 38.8°C in 5% CO2, 5% O2 in Nitrogen for cleavage rates determination. In order to allow comparison between the 6 males, 2 series of independent experiments (3 replicates) were performed including a homologous fertilization control (buck semen with proven efficiency) and repetition for one markhor male (male #4).

#### LOPU/IVF for markhor

LOPU/PIV sessions were carried out in 2012-2018 using oocytes collected from synchronized markhor females, stimulated by FSH (Folltropin-V, 100 mg/female) and anaesthetized (2 females per session in 2012, 2013 and 2017 and 3 females per session in 2018). In total, 9 ovum pick -up sessions were performed and oocytes were recovered from 7 distinct females.

Intravaginal sponges (20 mg FGA, MSD, Santé Animale) were inserted to markhor females on D0. On Day 8, IM injection of 0.3 mL of Estrumate (75 µg, MSD Santé Animale) was given. On D9, females received 100 mg Folltropin-V administered IM in 4 injections (1.87, 1.25 ,0.93 and 0.93 mL) at 12 h interval (48, 36 and 24 and 12 h prior to LOPU, respectively). The LOPU was performed on Day 11 under general anaesthesia induced by IM injection of Medetomidine/Ketamine (30µg/kg and 3mg/kg respectively) mixture. Animals were prepared for LOPU, performed as described previously (Locatelli et al., 2012). All follicles with a diameter of more than 2 mm were aspirated using a 20 G small ruminant needle from Watanabe Technologies (WTA, Brasil) connected to a controlled vacuum system (-35 mm Hg). Aspirated follicular fluid was collected in TCM-199 supplemented with Hepes (2.38 mg/mL), BSA (1 mg/mL), Heparin (10 IU/mL) and gentamycin (40 µg/mL). Abdominal wall was sutured, sponges were removed and anaesthesia was reversed by IM injection of atipamezole. Endotracheal intubation was removed at head-up of animal.

Aspirated cumulus oocyte complexes (COCs) were selected and washed as described previously ( Souza-Fabjan et al., 2014). The COCs were then allowed to mature in vitro in groups for 24 h in four well plastic dishes in 500 µL of TCM- 199 Mix medium ( Souza-Fabjan et al., 2014). After IVM, COCs were washed and fertilized with thawed semen as described upper using male #4 semen. Presumptive zygotes resulting from IVF were cultured in SOFaa supplemented with BSA and 10% FCS at 38.8°C, in humidified atmosphere either in 25µL microdroplets in 5% CO2, 5% O2 in Nitrogen or co-cultured in 500 µL well with ovine Oviduct Epithelial Cells (oOEC) in 5% CO2 in air.

#### Development of artificial insemination with frozen semen

Artificial inseminations set up for EAZA program were performed with 3 frozen semen samples from 2 genetically important males. Males were collected at Korkeasaari Zoo, Finland, either post mortem by epididymal retroperfusion in November 2017 (one male suffered from accidental injury during transportation) or electroejaculation performed under anaesthesia on male “ELMO” in 2018 and 2019. Electroejaculation and sperm freezing procedure were similar to description done upper. Synchronization of the female markhor 's cycle was coupled with intrauterine insemination with frozen/thawed semen performed by laparoscopy. Briefly, an estrus synchronization protocol (caprine method) was applied to 6 total markhor females (23-34 kg) in 6 attempts realized in 2017, 2018, 2020 and 2021. For each attempt, 3 females from the herd were recruited. Administration of progestagen treatment was performed with vaginal sponge (20 mg FGA, MSD Santé Animale) for 12 days coupled with the administration of 0,3 mL prostaglandins at day 8 (75 µg Estrumate, MSD Santé Animale) and 100 IU eCG (MSD Santé Animale) administered IM at withdrawal of sponge. Depending on insemination trials, AI procedure was performed at 52 h or 65 h following sponge removal on anesthetized females prepared for laparoscopy. Insemination took place under general anaesthesia induced by IM injection of medetomidine/ketamine (30µg/kg and 3mg/kg respectively) mixture. Abdominal region was shaved and prepared for laparoscopy and an endotracheal intubation was performed to maintain anaesthesia using isoflurane. Insemination was performed laparoscopically with 2 thawed french straws per female (2x50x10^6^ spermatozoa/mL) using ovine Transcap gun and Aspic (IMV technologies). Abdominal wall was sutured, anaesthesia was reversed by IM injection of atipamezole and endotracheal intubation was removed at head-up of animal.

## Results

### Semen recovery and viability at thawing

Ejaculates were successfully recovered during the breeding after 11 to 17 stimulations, generally at the first attempt. Volume of ejaculates was 1.06 ± 0.17 mL (m ± SEM) and sperm concentration was 4.57x10^9^ ± 0.32x10^9^ sptz/mL. Mean massal motility score assessed shortly after recovery was 4.1/5 and total motility ranged from 70 to 90%.

**Figure 1:**
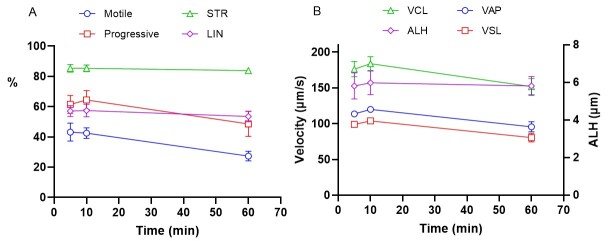
Sperm parameters determined by CASA evaluation at 5, 10 and 60 minutes post thawing for Tadjik markhor semen recovered by electroejaculation and frozen with caprine method (n= 6). A) Percentages of motile, progressive spermatozoa, percentages of straightness (STR) and linearity (LIN) of the movement. B). Velocity parameters (VSL, VCL, VAP and ALH) of motile spermatozoa. VSL: straight line velocity, VCL: curvilinear velocity, VAP: average path velocity, ALH: amplitude of lateral head displacement, LIN: linearity of the curvilinear trajectory (VSL/VCL × 100), STR : straightness (VSL/VAP × 100). Values represented are Mean ± SEM.

Mean sperm parameters for 6 males after cryopreservation and thawing assessed by CASA are represented in Figure 1. We observed good efficiency of the cryopreservation protocol on sperm survival since motility at thawing was 43% of total spermatozoa on average. Progressive motility was maximal 64.3% at 10 min after thawing over the 6 samples assessed. Mean VSL and VCL values observed, at 10 min, were 102.9 ± 2.0 and 183.5 ± 8.7 µm/s respectively.

### Heterologous in vitro fertilization

Heterologous fertilization rates (markhor semen x goat oocytes) were assessed by embryo cleavage for 6 males markhor over 2 series of 3 replicates (Table 1). Generally lower fertilization rates were observed at 24 hours post insemination (h*pi*) in the heterologous fertilization system when compared to the Control IVF (P < 0.05, Chi-square test). However, at 48 h*pi*, the overall efficiency of IVF with semen from markhor males #2, #3, #4 and #6 did not differ significantly from Control, as high cleavage rates were observed (P > 0.05). Cleavage rates observed with males #1 and #5 were significantly reduced when compared with other males (P < 0.05).

**Table 1:** Cleavage rates (24 and 48 hours post insemination observed after co -incubation of in vitro matured caprine oocytes (slaughterhouse ovaries) and spermatozoa from 6 markhor males. Control IVF was performed using selected domestic goat semen.

**Male samples**	**oocytes (n)**	**cleavage**		**cleavage**
		**24 hpi (%)**		**48 hpi (%)**
Control	135	38.5 ^a^		82.2^a^​
#1	120	0.8 ^b^		10^b^​
#2	154	13^c^​		77.9^a^​
#3	159	8.8^c^​		76.1^a^​
**#4**	**153**	**5.2 ^d^**		**74.5**^a^​
				
Control	192	14 ^a^		84 ^a^
**#4**	**179**	**4.6b**​		**80.4^a^**​
#5	191	2.1 ^b^		18.8^b^​
#6	181	9.3a​		71.8^a^​

Within a column, values with different letters differ significantly (P < 0.05).

#### LOPU/IVF for markhor

No complications were observed during the experiment for the animal enrolled in oocyte recovery. On average, it was possible to puncture more than 13 follicles per female per LOPU session, allowing the recovery of 8 oocytes per female per session (Table 2). After morphological evaluation, 87.5% of the oocytes were categorized as good quality oocytes and were considered suitable for use for IVP.

**Table 2:** Follicular response and oocytes recovered by LOPU at the time of oocyte collection by LOPU in markhor goats after progestagen treatment and follicular stimulation by FSH.

**n LOPU sessions (total markhor females involved)**	**mean number of follicle aspirated**	**mean number of oocytes recovered**
	**per female per LOPU session**	
9 (n=7)	13.63±1.53	8±0.74

Of the 9 *in vitro* production sessions, 2 were excluded from the IVP results for technical reasons (1 incubator failure and 1 contamination). The results of IVP in markhor are summarized in Table 3 on the basis of 7 replicates. In total 107 oocytes were submitted to IVM, IVF and IVC. It was possible to produce markhor embryos with oocytes recovered as 54.2% of oocytes subjected to IVF were cleaved at 48 h*pi*. Of the embryos produced *in vitro*, we note that almost a third developed to the morula and blastocyst stages after 8 days of culture. At day 8 *pi*, 19% of the embryos produced reached the blastocyst stage

**Table 3:** Rate of cleavage (48 hours post insemination) and development at the morula and blastocyst stages (8 days post insemination) observed during in vitro development in markhor.

**Replicates**	**Oocytes n**	**Cleaved 48 hpi %**	**Morula D8pi (% cleaved )**	**Blastocyst D8pi (% from cleaved )**	**Total Morula + blastocyst D8pi (% from cleaved)**
7	107	54.2%	12.1%	19.0%	31.0%

#### Development of artificial insemination with frozen semen

**Figure 2:**
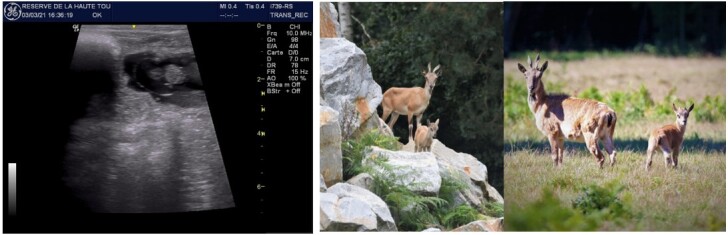
Pregnancy diagnosis in the Tadjik markhor at D35 post insemination and kids born unassisted after 163 and 161 days of gestation

Semen was recovered from cauda epididymis at necropsy from markhor male (transport injury) at Helsinki University. Unfortunately, freezing protocol was not optimally performed (conservation time, thermic stress of the samples) explaining lower viability at thawing than expected (20%/1,5/25% for motility/individual scoring/progressive, respectively). Despite poor quality of the sample and because of its important genetic value, AI was performed in a 52h fixed time protocol. None of the 3 females were pregnant at ultrasonography on Day 35. Procedure was repeated with frozen semen from second valuable male (ELMO 2) recovered in february 2018. Ejaculate was contaminated by urine at recovery quickly washed in KRPG and centrifugated. At thawing sperm scoring of the ejaculate was 35%/2.5/50%. None of the 3 females inseminated were pregnant at ultrasonography. New ejaculate was produced and frozen (ELMO 2#) in december 2019 (showing good quality at thawing (60%/3.5/50%). Of 6 females inseminated in 2020, none were pregnant at ultrasonography. Procedure was modified afterward to postpone fixed time AI at 65h post sponge removal (Table 4). Of the six females inseminated, 2 were pregnant at ultrasonography and the two females gave birth to 2 healthy males kids 163 and 161 days post insemination, respectively (Figure 2).

**Table 4:** Summary of the different inseminations carried out on the markhor female on the RZHT. Overall, six females were involved in the procedure.

**Year / month**	**AI time/ eCG**	**Female number**	**Semen /quality**	**pregnant**
2017/12	52h/100 IU	3	Helsinki 1/ very poor	0/3
2018/12	52h/100 IU	3	Helsinki 2/ poor	0/3
**2020/01**	**52h/100 IU**	**3**	**Helsinki 2#/good**	**0/3**
**2020/12**	**52h/100 IU**	**3**	**Helsinki 2#/good**	**0/3**
**2021/01**	**65h/100 IU**	**3**	**Helsinki 2#/good**	**1/3**
**2021/12**	**65h/100IU**	**3**	**Helsinki 2#/good**	**1/3**

## Discussion

The objective of the present study was to transpose the methods of sperm freezing from domestic goats to the Tadjik markhor (*Capra falconeri heptneri*) and to address the feasibility to develop IVP and artificial insemination using frozen semen. Semen of different adult markhor males were successfully recovered by electro-ejaculation then frozen using caprine methodology. Frozen semen showed good survival rates at thawing and good fertility assessed in heterologous fertilization system. In the present study, we applied LOPU/IVF for Tadjik markhor and reports the first successful blastocyst production *in vitro*. In an applied program, we also transposed successfully AI method with frozen/thawed semen to the Tadjik markhor.

The markhor is of major conservation interest within the EAZA institutions but certain limits were identified for the successful implementation of the EEP program (European Endangered species Program) for this species. On the one hand, the program is based on a limited number of founder individuals and on the other hand, the quality of the pedigree is poor as only about 30% of individuals are of known paternity origin making difficult to propose pertinent recommendation for genetic exchanges between conservation sites. Genetic studies are ongoing to identify individuals of high genetic value among captive subpopulations and to establish priority in genitor exchanges between zoos. In complement, assisted reproduction techniques would be particularly helpful reaching the objectives of EEP programs allowing both genetic diffusion and long term preservation of genetic.

Good quality ejaculates were recovered by electro-ejaculation and caprine methodology was efficient to preserve sperm parameters after freezing and thawing. Sperm recovery following electroejaculation and freezing in Bioxcell or Tris-egg yolk extenders has been previously reported (Bezjian et al., 2013) for Tadjik markhor semen. In the present study, ejaculates recovered during the breeding season showed increased main parameters (sperm concentrations, total motility) when compared to the study from Bezjian and collaborators. Differences in electro-ejaculation protocol, individuals or housing conditions may partly explain this phenomenon. Total motility observed at thawing was comparable, indicating different extenders can be employed to preserve viability of spermatozoa. However, progressive motility at thawing observed in the present study was improved, possibly indicating better preservation with the caprine milk-glycerol freezing when compared to Bioxcell or Tris-egg yolk methodologies.

Majority of frozen semen samples from markhor showed ability to fertilize slaughterhouse derived goat oocytes in heterologous IVF system. High cleavage rates (71-80%) were observed 48 hpi for 4/6 males evaluated underlining good preservation of fertilizing capacity after freezing. Heterologous IVF systems were previously employed to assess IVF conditions with frozen semen samples from wild species. Different heterologous systems were developed for semen evaluation such as oryx antelope semen x bovine oocytes (Roth et al., 1998), red and sika deer semen x bovine zona free oocytes (Comizzoli et al., 2001), Iberian ibex semen x goat oocytes (López-Saucedo et al., 2014) or ocelot or jaguar semen x cat oocytes (Santos et al., 2022; Stoops et al., 2007). The heterologous IVF system allows a double benefit in our conservation strategies for rare specimens. On the one hand, this approach allows additional evaluation of the viability of the semen upon thawing or the development of culture conditions for establishing the gametic interaction of the semen without using the oocytes of the species. On the other hand, the markhor/F1 goat hybrid embryos produced could ultimately allow creation of a population of recipient females whose physiologically close to that of the species of conservation interest. Thus, in a cryobanking approach, markhor embryos could be stored jointly with F1 markhor/goat hybrid embryos. Despite good viability and motility parameters, two males showed low fertilization rates in heterologous IVF system, evaluated at thawing. Further studies are required to clarify if some of the semen characteristic, the heterologous IVF system, or fixed IVF conditions may explain this phenomenon.

In the present study we report for the first time *in vitro* production of Tadjik markhor embryos using LOPU derived oocytes. After IVP, cleavage rates of 54% of oocytes was observed and almost 20% of IVP embryos were able to develop to the blastocyst stage after *in vitro* culture. Despite good quality of oocytes recovered after LOPU in markhor, fertilization rate was decreased when compared to heterologous IVF system with slaughterhouse oocytes, using the same male and IVF conditions. In a study performed in goat, comparing developmental competence between LOPU and slaughterhouse derived oocytes, such differences has been also reported (Souza-Fabjan et al., 2014). LOPU derived goat oocytes showed reduced cleavage rate after IVF but were fully developmentally competent as shown after parthenogenetic activation. Altogether, these data suggest reduced fertilization competence or specific requirement for LOPU derived markhor oocytes when compared with slaughterhouse derived goat oocytes. These techniques deserve to be perfected to enhance embryo yield and to allow the creation of embryo cryobank for *Capra falconeri heptneri.* Ongoing experiment has been designed to assess viability of IVP embryos using transfer on F1 hybrids recipient females.

Artificial insemination transposition was particularly difficult to achieve for markhor in zoo context. Restricted number of available females, will to minimize stress associated with handling procedure and main objective to obtain pregnancy with a given male limited us in the possibilities to precise physiology of the ovulation or to compare and adjust oestrus synchronization protocols. However, it has been possible to obtain 2 livebirths after performing AI with frozen semen for 6 females in a given condition. These encouraging results show AI will be possible to apply to this species for *ex situ* conservation programs but also highlight the need to better understand physiology of markhor.

## Conclusion

Assisted reproductive technologies such as AI with frozen semen or IVP of embryos can be successfully transpose to the markhor species. If further studies are required to optimize efficiency of estrus synchronization protocol and LOPU/IVP conditions for markhor, presents results illustrate that cryobanking of semen can be employed to efficiently preserve genetic of this species.

## References

[B001] Baril G, Chemineau P, Cognie Y (1993). Manuel de formation pour l’insémination artificielle chez les ovins et les caprins..

[B002] Bezjian M, Abou-Madi N, Kollias GV, Parks JE, Cheong SH, Beltaire KA (2013). Characterization and cryopreservation of semen from endangered markhor goats (capra falconeri heptneri) with evaluation of reproductive seasonality. J Zoo Wildl Med.

[B003] Comizzoli P, Mauget R, Mermillod P (2001). Assessment of in vitro fertility of deer spermatozoa by heterologous IVF with zona-free bovine oocytes. Theriogenology.

[B004] Corteel J-M, Baril G (1974). Viabilité des spermatozoïdes de bouc conservés et congelés avec ou sans leur plasma séminal : effet du glucose. Ann Biol anim Bioch Biophys.

[B005] Locatelli Y, Hendriks A, Vallet J-C, Baril G, Duffard N, Bon N, Ortiz K, Scala C, Maurel MC, Mermillod P, Legendre X (2012). Assessment LOPU-IVF in Japanese sika deer (Cervus nippon nippon) and application to Vietnamese sika deer (Cervus nippon pseudaxis) a related subspecies threatened with extinction. Theriogenology.

[B006] Locatelli Y (2024). L’assistance médicale à la procréation au service de la biodiversité. Médecine de La Reproduction..

[B007] López-Saucedo J, Paramio MT, Fierro R, Izquierdo D, Catalá MG, Coloma MA, Toledano-Díaz A, López-Sebastián A, Santiago-Moreno J (2014). Sperm characteristics and heterologous in vitro fertilisation capacity of Iberian ibex (Capra pyrenaica) epididymal sperm, frozen in the presence of the enzymatic antioxidant catalase. Cryobiology.

[B008] Roth TL, Weiss RB, Buff JL, Bush LM, Wildt DE, Bush M (1998). Heterologous in vitro fertilization and sperm capacitation in an endangered african antelope, the scimitar-horned oryx (oryx dammah). Biol Reprod.

[B009] Santos MVO, Silva HVR, Bezerra LGP, de Oliveira LRM, de Oliveira MF, Alves ND, da Silva LDM, Silva AR, Pereira AF (2022). Heterologous in vitro fertilization and embryo production for assessment of jaguar (Panthera onca Linnaeus, 1758) frozen-thawed semen in different extenders. Anim Reprod.

[B010] Souza-Fabjan JMG, Locatelli Y, Duffard N, Corbin E, Batista RITP, de Figueirêdo Freitas VJ, Beckers JF, Mermillod P (2016). Intrinsic quality of goat oocytes already found denuded at collection for in vitro embryo production. Theriogenology.

[B011] Souza-Fabjan JMG, Locatelli Y, Duffard N, Corbin E, Touze J-L, Perreau C, Beckers JF, Freitas VJ, Mermillod P (2014). In vitro embryo production in goats: slaughterhouse and laparoscopic ovum pick up-derived oocytes have different kinetics and requirements regarding maturation media. Theriogenology.

[B012] Stoops MA, Bond JB, Bateman HL, Campbell MK, Levens GP, Bowsher TR, Ferrell ST, Swanson WF (2007). Comparison of different sperm cryopreservation procedures on post-thaw quality and heterologous in vitro fertilisation success in the ocelot (Leopardus pardalis). Reprod Fertil Dev.

